# Echoes of the Month

**Published:** 1924-02

**Authors:** 


### ECHOES OF THE MONTH.

### DIET AND CANCER.

The medical correspondent of the *Observer* has been comparing "the dietaries of several contrasted nations, in relation to the hypothesis that the notorious increase of cancer in civilised communities may be related to their unnatural habits of feeding. In four preceding years I have seen, very extensively, the practices of the United States, and I am still of the opinion, formed in 1919, that overfeeding is the one conspicuous sin against physiological law committed in that country. This over-feeding is very general, and often very gross. During the year now closing I have partaken of hospitality, first, in three Northern countries, Denmark, Switzerland and Finland. It is not open to question that feeding is excessive in Denmark and Sweden. This is notably true of proteins—typically represented by meat. It needs only a brief stay in Switzerland, a land unhappily conspicuous for its very high cancer death-rate, to observe the large quantities of protein consumed. Even allowing for possibly inferior diagnosis in respect of some forms of cancer, there seems to be no doubt that the malignant death-rate is distinctly lower in Italy, for instance, than in the northern countries. Indeed, the generalisation has been made that the cancer death-rate rises as one passes from the tropics northwards. The assertion has also been made that where malaria abounds cancer does not, and the suggestion has been made that the malarial poison may be antagonistic to malignant cells."

### INTELLIGENCE AND SIZE OF THE HEAD.

Lecturing on "Anthropometric Analysis" at Aberdeen University, Professor Reid said that during the last twenty-seven years anthropometric observations had been made by himself and his assistants upon students attending the University. There was a similarity in stature, shape of head, shape of face, shape of nose and colour of hair between the students who were native to the North-East of Scotland and the natives of the districts of Norway. The length of a man's head depended to a certain extent upon his stature, but the breadth of his head was unaffected by his stature. The cephalic index tended to vary inversely with the stature, or, to put it in popular language, the longer a man's head was the taller he was likely to be. The conclusion arrived at with reference to the relation of cranial capacity to intelligence confirmed the opinion already arrived at by Pearson and Macdonell that the anthropometric examination of a person's head was no guide to the intelligence of its owner.

### A PARIS DOCTOR'S LIBRARY.

Dr. Ripault, of Montmartre, says the Paris correspondent of the *Times*, has decided to sell his famous collection of books, valued at £10,000 at present rate of exchange. He possesses books which belonged to Henry IV., Louis XIV., Louis XV., Louis XVI., Napoleon, Louis Philippe, Marie Antoinette, Richelieu and Mazarin. Another part of his collection was made on the principle of obtaining original editions of great works. Thus there are first editions of Copernicus, Tycho-Brahe, Galileo, Newton's Treatise on Universal Attraction, Papin's book on the application of steam-power, Galvani on electricity, Harvey on the circulation of the blood, and various works of Lamarck, Cuvier, Darwin and Claud Barnard. Dr. Ripault comes of a family which has contained famous book collectors. His great-grandfather, Louis Ripault, was librarian to Napoleon, and his great uncle, Louis Ripault-Desormeaux, was librarian to the last Prince de Condé of the old line.

### REMINISCENCES OF SIR DYCE DUCKWORTH.

Sir Dyce Duckworth, one of Liverpool's distinguished sons, recently gave an interesting account of his earliest recollections of his native city to the Literary and Philosophical Society of Liverpool. "My first railway experience," he said, "was a journey to and from Manchester in 1848. We were warned to hold on tightly when the train stopped for fear of being thrown off our seats. We were drawn up to Edge Hill by a rope, no engines being sent down to Lime Street until many years after. Our greatest joy was to be on the various ferryboats, to see the Cunard steamers receiving their mails, firing their guns as they left their anchorage, and again when the mails were on board. The rival Collins Line steamers also fired as they shipped their mails, till one day a man on the Prince's Pier was hit by a cartridge from one of their guns and killed. This put a stop to the proceeding for both firms. In my early days we had the advantage of securing British fresh beef, mutton and bacon. The general supply of milk was then certainly dangerous, owing to ignorance and uncleanness, and was often a source of scarlet fever. The drainage of our houses was very insanitary. Rats readily entered houses from the horrible old midden system. The streets of the town were laid with large, water-worn stones. Later on we were supplied with cut stones, obtained by disfiguring the beautiful slopes of Penmaenmawr, and now we have come to wood and tar and gravel."

### OPERATIONS ON CONSCIOUS PATIENTS.

For the first time in this country, major operations are being performed with the use of a drug which does not deprive the patient of consciousness. At one of the London hospitals recently, a man had two-thirds of his stomach removed, and, although he was conscious all the time, he did not feel the slightest pain. So well was he after the operation that three hours later he asked if he could have a smoke. The great advantages of the new method are that there are none of the unpleasant after-effects which accompany the use of a general anæsthetic, and there is an entire absence of shock, because the nerves at the part where the tissues are cut refrain from acting, and so do not send any messages of pain to the brain. It can be used in the case of patients suffering from heart weakness, where a general anæsthetic would be impossible. The drug is injected into the back in the case of abdominal operations, and in the side of the neck in the case of cancer of the tongue, throat or lip. The patient does not experience any unpleasantness, as his eyes are covered and his ears plugged.

### HEALTH PRIZES FOR AMERICAN TOWNS.

An important co-operative enterprise of the United States Public Health Service is the establishment of a joint office with the American Public Health Association where health officers and others can obtain the latest scientific information on municipal health department practice. The establishment of an office of administrative public health in Baltimore is the next step in its scheme of co-operation. The data in the Baltimore office and the services of a medical officer will be available at all times to any town that wants guidance in developing its local health work. A system of classifying towns as to their effectiveness in health work will be devised as a basis for determining the three prizes that are to be awarded for the most complete community health service. The awards are offered by the American Public Health Association, and will be presented at its fifty-third annual meeting at Detroit this year.

### A NATIONAL ASSET.

The view expressed by Mr. J. S. Alford in his presidential address to the Institute of Sanitary Engineers that "a continuance of the decline in the death-rate is both undesirable and unlikely," is not being allowed to pass unchallenged by the Ministry of Health. "Longer lives are not a burden to the nation," said a Ministry of Health official to a Press representative. "The march of hygienic development has resulted in a race of men over 50 who are more efficient and of more general value to the nation than the average run of men of fifty, of twenty and thirty years ago." Mr. Alford also stated that the more intelligent classes appeared to be diminishing relatively to the less intelligent. The retort of the Ministry of Health to this is: "There has never been a higher level of intelligence in the country than at present"; and Sir George Newman's report on the further saving of infant lives was quoted in support of the argument that the figures implied a better physical and a more enlightened understanding of personal and public hygiene which must contribute to the well-being of men over 50 and 60 years of age. " Instead of being a burden to the nation," continued the Ministry official, " men reared under the present conditions of scientific instruction are a great asset to the nation when they come to middle age."

### X-RAYING TUTANKHAMEN.

We learn (says the *Lancet*) that Dr. D. E. Derry, Professor of Anatomy at the Government Medical School in Cairo and a well-known anthropologist, has been charged with the examination of the mummy of Tutankhamen. Calcified ova of bilharzia have before now been found in mummified kidneys, and the calcification of the arteries seems to have been one of the commonest of diseases in the time of the Pharaohs, affecting both young and old. What else the examination may reveal is a matter for intelligent speculation, but nothing is likely to miss the combined efforts of anatomist, anthropologist and radiologist.

### HOPES AND FEARS FOR THE MARRIED.

The Registrar-General for Scotland states that expectation of female life at birth is now 56.35 years as against 42.05 in the year 1871, 47.47 in the period 1891-1900, and 53.18 in 1911. The expectation of female life was throughout the entire period of life better than the male expectation. The rate of mortality among unmarried males was found to be in excess of that among married males, and the expectation of life among married males was correspondingly and materially greater than among the unmarried. This might be influenced not only by conditions of marriage but by other conditions, and more especially by a process of selection previous to marriage—the more robust and healthier joining the married ranks, the less robust remaining among the unmarried. In marked contrast to the male tables, the differences of expectation shown between the unmarried female and married female tables were small, and though generally in favour of the married, were at certain periods in favour of the single. The fact that the rate of mortality among married females aged 25 to 40 was in excess of that of the unmarried was suggestive of this rate being affected by the risk of puerperal death among married women.

### SEVEN THOUSAND AMERICAN HOSPITALS.

Whereas in 1873 there were but 149 hospitals in the United States and its possessions, in 1923 there are 7,095, and whereas in 1873 there were but 35,453 patient beds, in 1923 there are 792,069. Furthermore, a distribution of the hospitals in the United States, not including its possessions, shows that 1,546 are in towns having a population of 100,000 and over, 446 in towns of 50,000 to 100,000, 532 in towns of 25,000 to 50,000, 1,001 in towns of 10,000 to 25,000 and 3,366 in towns of 10,000 and under.

### EMBALMING AND MEDICINE.

The practice of embalming the dead in ancient Egypt, said Professor G. Elliot-Smith in a lecture to the Egyptian Exploration Fund, was interesting for other reasons than as a strange and gruesome custom, which had made it possible for us to gaze upon the actual faces of the dominant personalities of the civilisation of thirty centuries ago. The connection of mummification with the development of medical science and practice was specially intimate. It familiarised the embalmers with certain aspects of the structure of the human body, and—what was far more important—prepared the minds of the people to tolerate the practice of dissection, and acquainted them with the antiseptic properties of certain vegetable and mineral substances that afterwards played an important part in the treatment of disease. It was regarded not only as a means of prolonging indefinitely the existence of the dead person, but also as a device for conferring life upon him; in other words, of preventing the cessation of his vital activities. Thus its aim was brought into essential agreement with that of the primitive physician, namely, to combat disease—which was interpreted as a reduction of vitality—by conferring upon the patient additional vital substance.

